# A Personalized Autism Diagnosis CAD System Using a Fusion of Structural MRI and Resting-State Functional MRI Data

**DOI:** 10.3389/fpsyt.2019.00392

**Published:** 2019-07-04

**Authors:** Omar Dekhil, Mohamed Ali, Yaser El-Nakieb, Ahmed Shalaby, Ahmed Soliman, Andrew Switala, Ali Mahmoud, Mohammed Ghazal, Hassan Hajjdiab, Manuel F. Casanova, Adel Elmaghraby, Robert Keynton, Ayman El-Baz, Gregory Barnes

**Affiliations:** ^1^Bioimaging Lab, Bioengineering Department, University of Louisville, Louisville, KY, United States; ^2^Department of Electrical and Computer Engineering, Abu Dhabi University, Abu Dhabi, United Arab Emirates; ^3^Department of Biomedical Sciences, University of South Carolina, Greenville, SC, United States; ^4^Computer Engineering and Computer Science Department, University of Louisville, Louisville, KY, United States; ^5^Bioengineering Department, University of Louisville, Louisville, KY, United States; ^6^Department of Neurology, University of Louisville, Louisville, KY, United States

**Keywords:** structural magnetic resonance imaging, functional magnetic resonance imaging, autism, personalized diagnosis, Computer-Added Diagnostics (CAD) systems, machine learning

## Abstract

Autism spectrum disorder is a neuro-developmental disorder that affects the social abilities of the patients. Yet, the gold standard of autism diagnosis is the autism diagnostic observation schedule (ADOS). In this study, we are implementing a computer-aided diagnosis system that utilizes structural MRI (sMRI) and resting-state functional MRI (fMRI) to demonstrate that both anatomical abnormalities and functional connectivity abnormalities have high prediction ability of autism. The proposed system studies how the anatomical and functional connectivity metrics provide an overall diagnosis of whether the subject is autistic or not and are correlated with ADOS scores. The system provides a personalized report per subject to show what areas are more affected by autism-related impairment. Our system achieved accuracies of 75% when using fMRI data only, 79% when using sMRI data only, and 81% when fusing both together. Such a system achieves an important next step towards delineating the neurocircuits responsible for the autism diagnosis and hence may provide better options for physicians in devising personalized treatment plans.

## Introduction

Autism spectrum disorder (ASD) is a neuro-developmental disorder that has three main associated characteristics (1): i) social functioning disorders, ii) communication impairments, and iii) restricted and repetitive behaviors (RRBs). In many previous research projects, correlation was reported between autism and both anatomical abnormalities and functional activation abnormalities. For studying anatomical abnormalities, the most commonly used imaging modality is structural MRI (sMRI) (2), while functional MRI (fMRI) is the most commonly used modality for studying brain activation (3).

The relationships between MRI parameters and the autism diagnosis play a key role in defining the impaired neurocircuits in an individual ASD subject. When studying anatomical sMRI, there are two main categories of features, and each study either uses features driven from one of them or a combination of both i) shape features and ii) volumetric features. With regard to volumetric analysis, Courchesne et al. (4) conducted a study on 60 autistic and 52 typically developed individuals (ages between 2 and 16 years) to explore the anatomical abnormalities in cerebral and cerebellar volume of autistic brains with 50% of the autistic participants being aged 5 or more years and 50% between 2 and 4 years old. In the age group between 2 and 4 years old, 90% of the participants were found to have brain volumes larger than normal. This result reinforced the hypothesis that the brain volume in autistic infants was larger in size than in typically developed infants. This hypothesis was also supported by results in the study of Hazlett et al. (5), where 51 autistic and 25 typically developed individuals (ages between 1.5 and 3 years) were examined and it was found that the cerebellar white matter volume in autistic subjects between 2 and 4 years old were larger than normal size. Geschwind and Levitt (6) also emphasized the same assumption during infancy interval, but found additionally that the cerebral hemisphere can remain enlarged during adulthood. Meanwhile, other investigators (7–9) suggested that the main areas of the brain with enlarged white matter and gray matter were the frontal, temporal, and parietal lobes (ages between 2 and 12 years). A voxel-based morphometry (VBM) study was conducted by Toal et al. (10) to study the brain anatomy of both autistic and typically developed adults (mean age is 32 years with 9 years standard deviation), and found that the brain of the autistic individuals had significant increased gray matter involving both the frontal and temporal lobes.

Instead of studying the cortical volume (CV) as a single parameter, and based on the fact that CV is the product of two parameters, cortical thickness (CT) and surface area (SA), Ecker et al. (11) analyzed these three parameters together in order to attain a more insightful observation about anatomical abnormalities in the autistic brains (age mean is 26 years with 7 years standard deviation). This study observed differences in the three parameters (CV, CT, and SA) between the two groups with the CT in autistic subjects being significantly larger than that in typically developed individuals in the frontal lobe regions, while SA in the orbitofrontal cortex and posterior cingulum in autistic subjects was less than that in the typically developed. Using the same three parameters, a more comprehensive study by Haar et al. (12) was conducted (ages between 6 and 65 years). While more detailed results were obtained from this study, the results were still in line with previous studies and anatomical hypotheses. Specifically, in autistic individuals, larger ventricular volumes, smaller corpus callosum volume (central segment only), and several cortical areas with increased thickness were detected. Another approach for studying anatomical abnormalities in autistic subjects was to study the longitudinal changes in CT (13) (ages between 3 and 36 years), which allows the identification of specific regional differences in the CT. In their study, Zielinski et al. (13) discovered that the most significant differences in CT between autistic and typically developed individuals of the same mean age was in the bilateral inferior frontal gyrus, pars opercularis, pars triangularis, right caudal middle frontal, and left rostral middle frontal regions. Other studies addressed different brain regions. For example, autistic subjects displayed larger amygdala than normal subjects (14). Waiter et al. (15) (age mean is 15.4 years with 2.24 years standard deviation) examined the areas believed to be responsible for the social cognitive functions: in particular, 1) facial recognition (right fusiform gyrus), 2) perception and eye gaze (superior temporal gyrus), and 3) mental state attribution (anterior cingulate and superior temporal sulcus). In these areas, a significant increase in gray matter was observed. Another VBM study was performed by Salmond et al. (16) (ages between 8 and 18 years) to examine the cerebellum, fusiform gyrus, and frontal cortex. Similarly, increased gray matter volume was observed in the regions of the cerebellum in the participants located near the high functioning end of the autism spectrum, which is consistent with the anatomical hypothesis that it relates increased brain size to autism.

Another major approach explored for discriminating between autistic and typically normal developed brains was shape-based analysis of sMRI. As a quantitative measure for shape analysis, the gyrification index (GI) (17) was used by Hardan et al. (18) (age mean is 12.7 years with 2.2 years standard deviation). GI is a measure of cortical folding that is calculated as the ratio between total contour length and outer contour length from coronal sMRI slices. The GI in the left frontal area was noticed to be larger in autistic children and adolescents than corresponding typically developed children and adolescents. In this study, the GI decreased with age in autistic subjects but not in typically developed subjects. In line with the increased GI finding reported by Hardan et al. (18), Wallace et al. (19) (age mean is 16.7 years with 2.8 years standard deviation) also reported increased gyrification in the bilateral posterior cortices in autistic subjects. Furthermore, a positive correlation between vocabulary knowledge and gyrification in the left inferior parietal cortex in typically developed individuals was noted while no correlation was found for autistic subjects. One of the most commonly used shape-based analysis techniques was folding analysis. For example, Awate et al. (20) (ages between 7.5 and 31 years) used six folding measures for cortical curvature analysis between both groups (autistic and typically developed). The folding measures used were i) intrinsic curvature index, ii) mean curvature norm, iii) convexity ratio, iv) isoperimetric ratio, v) shape index *S*, and vi) curvedness. The Awate et al. (20) study found increased folding in the ASD frontal, parietal, and temporal lobes when compared to typically developed individuals. This increased folding was more prominent in children than in adults. A more recent study by Katuwal et al. (21) also addressed the curvature abnormalities using seven features extracted from a reconstructed brain mesh. The features used were i) Gaussian curvature, ii) mean curvature, iii) folding index, iv) thickness, v) thickness standard deviation, vi) SA, and vii) volume. Another study by Nordahl et al. (22) (ages between 7.5 and 18 years) addressed the cortical shape abnormalities in both high-functioning and low-functioning ASDs plus TDs using surface-based morphometry. Sulcal depth was used as a quantitative measure to analyze morphological abnormalities. For the low-functioning autistic subjects, the abnormalities in sulcal depth were mainly noticed in the anterior insula and frontal operculum in addition to shape abnormalities in the inferior frontal gyrus. The same abnormalities were noticed in high-functioning autistic patients, but with relatively smaller size. They were centered near the parietal operculum and ventral postcentral gyrus. Sulcal depth differences were also reported by Dierker et al. (23) (age mean is 11.4 years with 1.9 years standard deviation), in which differences in sulcal depth were noted in the anterior insula and temporoparietal junction between the two groups. The areas having the most significant abnormalities were the frontal and temporal areas, particularly from social and language regions, which were highly implicated in autism. Brain shape differences between autistic and typically developed individuals were explored by Ecker et al. (24) (ages between 18 and 43 years), where GI in gray matter was studied. The experimental outcome was a prominent increase in gyrification around the left pre- and post-central gyrus in autistic individuals.

Regarding fMRI analysis, there are two major types of experiments to examine brain functional activity: i) resting-state fMRI (RfMRI) and ii) task-based fMRI (25) (age mean is 24 years with 10 years standard deviation).

In Just et al. (26), the underconnectivity theory was first proposed. This theory states that ASD is due to both cognitive and neurobiological disorders. The cognitive disorder mainly appears as reduced synchronized brain activity in integrative processing demanding tasks, for example, forming a sentence from a set of words (27) (age mean is 27.1 years with 11.9 years standard deviation).

Researchers conducted different studies to study the brain connectivity using task-based approaches. For example, less activation in the left dorsolateral prefrontal and inferior parietal areas was identified, and more activation was reported in the right occipital (visuospatial) areas and bilateral superior parietal using a figures task experiment in Damarla et al. (28) (age mean is 19 years with 5.5 years standard deviation). In Weng et al. (29) (age mean is 14.36 years with 1.7 years standard deviation), the response to facial expressions was studied, where autistic individuals were reported to have higher activation in the amygdala, ventral prefrontal cortex, and striatum. Another example of using task-based experiments is the rewards task, where subjects are given either monetary or social reward and their brain activity in response to this reward is recorded (30, 31) (age mean is 12.3years with 1.76 years standard deviation). In Dichter et al. (32), less activation in the right nucleus accumbens and more activation in left midfrontal and anterior cingulate gyrus were reported in ASDs than in TDs in response to social and monetary rewards. Another study by Cox et al. (33) (age mean is 24.11 years with 4.16 years standard deviation) supported less connectivity in autistic subjects in response to rewards.

To study the alterations in connectivity between TDs and ASDs, Deshpande et al. (34) (age mean is 21.14 years with 1 year standard deviation) applied a machine learning algorithm based on a multivariate autoregressive model trying to find the most logical end to a story shown to them.

In the study by Itahashi et al. (35) (age mean is 31 years with 8 years standard deviation), researchers found that functional connectivity of ASDs is less than that of TD subjects. These results were also supported by Alaerts et al. (36) (age mean is 13.7 years with 4.64 years standard deviation), which is providing more evidence for the underconnectivity theory.

In Rausch et al. (37) (age mean is 16.23 years with 3.218 years standard deviation), reduced functional connectivity in visuospatial and superior parietal areas was reported on ASDs when compared to TDs. Also in another study by Tyszka et al. (38) (age mean is 27.4 years with 2.4 years standard deviation), reduced connectivity was reported in local areas of both the frontal and temporal cortex, but no global abnormalities were detected. In Plitt et al. (39) (age mean is 17.5 years with 5.5 years standard deviation), dysfunction in the functional networks was reported, and this dysfunction was more obvious in social information processing related networks. The altered connectivity result was also supported by Di Martino et al. (40), where both hypoconnectivity and hyperconnectivity were reported in ASD circuits.

Not only underconnectivity was reported for ASDs in the previous studies. A study by Hahamy et al. (41) (ages between 18 and 44 years) reported alterations in functional connectivity patterns, where the interhemispheric connectivity analysis in autistic subjects showed areas of decreased connectivity while other areas showed increased connectivity compared to healthy control subjects. The hyperconnectivity was also reported in autistic children in Supekar et al. (42) (ages between 7 and 13 years), where autistic children with more severe social dysfunction were found to be functionally hyperconnected.

In addition to reporting global differences between ASDs and TDs, resting-state connectivity patterns demonstrated promising results in diagnosing many diseases, e.g., Alzheimer’s disease (43), schizophrenia (44) (age mean is 35.9 years with 13.5 years standard deviation), and autism. For example, the approaches in Kim et al. (44) achieved high accuracy in schizophrenia diagnosis.

A deep neural network and functional connectivity analysis have been used in the recent study by Heinsfeld et al. (45) and Dvornek et al. (46) for autism diagnosis where the functional connectivity correlation matrix was the input to the classification network.

The heterogeneity of autism among individuals according to symptoms and severity has raised the need for a more personalized approach to predict and analyze the behavior and functionality of each autistic subject. Hence, we could then design an optimum treatment plan for every autistic subject. In this study, we aim to answer two main research questions: i) Can fMRI and sMRI be used for autism diagnosis in an objective way? ii) Are fMRI and sMRI features associated with ADOS scores? The hypothesis of this study is that combined sMRI and fMRI parameters are more likely to correlate more closely with behavior and yield high diagnostic accuracy, sensitivity, and specificity. The proposed system uses machine learning to define global and local features of ASD regardless of age or gender. Finally, we again analyze our results to be sure that it fits nicely within research domain criteria (RDoC)-defined neurocircuits related to ASD. Such criteria are important for the generalization of this model to highly heterogeneous ASD populations that present to the physician’s office.

## Materials and Methods

In this study, both fMRI and sMRI data are obtained from the National Database of Autism Research (NDAR). The data for both experiments are obtained from a single study (NDAR study ID 2021). Imaging data provided by NDAR are fully anonymized and they are linked with other records (diagnostic, behavioral, demographic, etc.) using a unique identifier, the NDAR globally unique identifier (GUID). The total number of subjects used is 185 subjects. The selected data were collected at George Washington University. All the selected subjects have both high-resolution T1-weighted structural images and RfMRI images (7 subjects out of the 185 have corrupted RfMRI imaging files, so they were included in the sMRI analysis and excluded from fMRI analysis and from sMRI–fMRI modalities fusion). Out of the used 185 subjects, 61 subjects have autism diagnostic observation schedule (ADOS) reports.

All neuroimages were produced by a Siemens Magnetom TrioTim with a 3-T magnet. Structural scans used an MPRAGE pulse sequence with TR = 2,530 ms, TE = 3.31 ms, TI = 1,100 ms, and flip angle 7°. Volumes were acquired in 3D with isotropic 1-mm voxel spacing. For the functional scans, they have TR = 2,000 ms, TE = 30 ms, and flip angle 90° in a two-dimensional acquisition sequence to produce images with 3-mm pixel spacing and 4-mm slice spacing. Time to acquire 33 coronal slices spanning the entire brain was 2.01 s, and the resting-state data were recorded for approximately 6 min.

### sMRI Experiment

In this experiment, both morphological and volumetric features are extracted and studied. Prior to extracting any of these features, there are some mandatory data preprocessing steps to be applied followed by the segmentation of the brain cortex. These preprocessing steps could be summarized as follows:

#### Brain Data Preprocessing

The preprocessing is a vital requirement to remove the variability between subjects that may stem from data acquisition, different scanners, artifacts, or partial volume effects. Moreover, the preprocessing step removes non-brain tissues such as skull. The following steps are applied to preprocessing sequentially.

1. Intensity normalization (47): In this step, intensity non-uniformities are corrected using a non-parametric model. It does not require any prior knowledge about existing tissue classes in the image.
2. Brain extraction and skull stripping (48): In this step, an algorithm combining both watershed algorithm and deformable surface model is used for skull stripping. The used algorithm starts by localizing a voxel belonging to the white matter, creating local minimum in the white matter, and then applying watershed algorithm with a pre-flooding height. This creates an initial estimate about the brain volume. To overcome any inaccuracies that might lead to cortical surface erosion, a deformable surface model is then applied. This allows the integration of geometric constraints into the skull stripping process.

#### Brain Segmentation and Area Labeling

The atlas-based brain segmentation task (49, 50) is formulated as a joint model using the given atlas and an affine transformation with 12 degrees of freedom, ω = *f* that maps the input volume to the atlas domain.

Let **R** = {*r* = (*x, y, z*): 0 ≤ *x* ≤ *X* − 1, 0 ≤ *y* ≤ *Y* − 1,0 ≤ *z* ≤ *Z* – 1}; **Q** = {0, 1,…, *Q* − 1}; and **L** = {0,…,*l*} denote a finite arithmetic lattice of the size *XYZ* supporting gray scale images and their region (segmentation) maps, a finite set of *Q* integer gray values, and a labeled set of objects (“0”), non-brain tissue (“1”), cerebrospinal fluid (CSF) (“2”) for gray matter, and so on. Let *g* = {*g* *_r_*: *r* ∈ **R**; *g* *_r_* ∈ Q} and *m* = {*m* *_r_*: *r* ∈ **R**; *m* *_r_*: ∈ **L**} be a gray-scale image having values from **L**, i.e., **m**: **R** ↦ L, respectively.

First, the brain atlas, **A** = {*a* *_i_* = (*g* *_i_*, *m* *_i_*): *i* = 1, 2,…, *N*}, contains 3D MRI scans of different brains and their manually labeled volumes. Given the atlas function, *f*, that co-aligns *a* *_i_* to the atlas domain (preselected template). This atlas is constructed in such a way that retains each anatomical label information at each voxel. The prior probability for each label *m* to occur at atlas location *r* is:

(1) $$P(m(r)=m)\approx \frac{\#\text{ of times label }m\text{ occurred at location }f(r)}{\#\text{ of voxels that map to }r\text{ in the training set}}$$

Since each location *r* can be mapped to different labels, the intensity distribution of each label *m* at *r* is modeled as a Gaussian distribution. The mean and variance of such distribution are calculated as:

(2) $$\mu_{m}(r))=\frac{1}{N}\sum_{i=1}^{N}g_{i}(f(r))$$

where g*_i_* are the set of *N* images for which label *m* occurs at location *f*(*r*) in the corresponding manually labeled image *S* *_i_*. The variance for label *m* at location *r* is given by:

(3) $$\sigma_{m}{\left(r\right)}^{2}=\frac{1}{N}\sum_{i=1}^{N}{\left(g_{i}(f(r))-\mu_{m}(r)\right)}^{2}$$

Having both prior information and conditional probability for each class at each atlas location, the segmentation problem for a new input subject, given its affine transformation, ω, for the atlas domain is modeled using MAP estimate:

(4) P(m|g,ω)=P(g|m,ω)P(m)

with the assumption that the noise is independent at *r*, *P*(**g**|**m**, *ω*) can be written as:

(5) $$P(g|m,\omega )=\prod_{r\in R}P(g(\omega_{r})|m(r))$$

Using the atlas information, Eqs. 2 and 3, the conditional probability for each label at each voxel is given by:

(6) $$P(g(\omega_{r})|m(r)=m)=\frac{1}{\sqrt{2\pi \sigma_{m}(r)}}\exp(-0.5\sigma_{m}{\left(r\right)}^{-2}(g(\omega_{r})-\mu_{m}(r))$$

To get the prior probability (*P*(**m**)) for Eq. 4, a Markov random field model is used to encode the label’s relationship as a function of location within the brain in addition to the local direction. Taking into account 6 voxels in the positive and negative cardinal directions at each location in the atlas space, the *P*(**m**) is expressed as:

(7) $$P(m)=\prod_{r\in \rho }P(m_{r})\prod_{i=1}^{m}P(m(r_{i})|m(r),r_{i})$$

where ρ is the neighborhood system of *r*. The values of *P*(**m**(*r*)) are computed and stored in the atlas using Eq. 1. The spatial relationship between different labels is encoded in *P*(**m**(*r* *_i_*)|**m**(*r*), *r* *_i_*). However, for simplicity and computation efficiency, the MAP estimate assumes *P*(**m**(*r* *_i_*)|**m**(*r*), *r* *_i_*) as uniform as no labels have been assigned yet. After obtaining the initial segmentation, it is sequentially updated using iterated conditional modes (ICMs) algorithm. For more details about the segmentation algorithm, the reader is referred to Sled et al. (47).

After completing the preprocessing and the segmentation steps, the following steps are applied for 3D surface reconstruction and brain parcellation to an anatomical atlas from the segmented volume.

1. Tessellation of the gray–white matter boundary (51, 52): In this step, the spherical topology of the surface is accurately corrected. The used technique constructs a mapping between the original surface onto a sphere. Topological defects are detected as the minimal nonhomeomorphic regions. Each topological defect is then corrected by opening and sealing along the set of non-separating loops.
2. Surface inflation and spherical atlas registration (53, 54): In order to establish a spherical-based cortical surface, three steps are applied: i) inflate the cortical surface to visualize hidden structures in the sulci, ii) cut and flatten the entire hemisphere, and iii) parameterize using a sphere. The parameterized surface is then used to create a spherical surface-based coordinate system. To define such coordinate system, the average folding pattern of a large population is used as an atlas. Each individual subject is then aligned to this atlas.
3. Cortical surface parcellation to the Desikan–Killiany (DK) atlas (55): In this step, each hemisphere is parcellated into 34 cortical labels.

**Figure 1:**
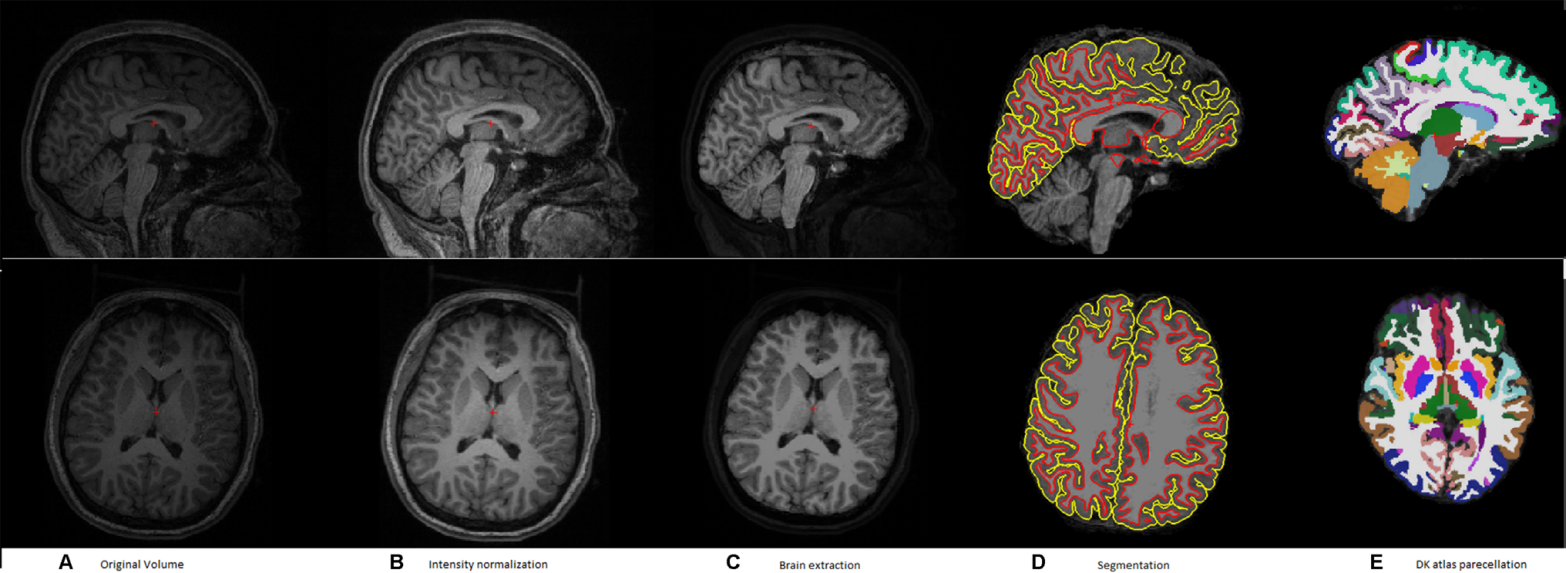
A typical example of the pipeline applied to an input volume to prepare it for feature extraction. **(A)** Original volume, **(B)** intensity normalization, **(C)** brain extraction, **(D)** segmentation, and **(E)** DK atlas parcellation.

**Figure 1** shows a typical sample of (a) an original volume, (b) intensity normalization, (c) brain extraction, (d) segmentation, and (e) DK atlas parcellation

After completing the above steps, eight features are calculated for each of the 34 hemisphere areas. The eight calculated features for each DK atlas area are as follows:

1. Surface area (*A*).
2. Volume (*V*).
3. Average thickness (*T*).
4. Standard deviation of the thickness (*T*).
5. Average of mean curvature (MCI), defined as: $MCI=\frac{K1+K2}{2}$, where *K*1, *K*2 are the two principle curvatures calculated at each vertex.
6. Average of Gaussian curvature (*K*), defined as: *K* = *K*1 * *K*2.
7. The average intrinsic curvature index (ICI), defined as: *ICI* = *MAX* (*K*, 0).
8. The average folding index (FI), defined as: *FI* = *ABS* (*K*1) * (*ABS* (*K*1) – *ABS* (*K*2)).

In this study, we used the publicly available and widely used brain MRI analysis software FreeSurfer pipeline, available at: http://surfer.nmr.mgh.harvard.edu/ for all preprocessing and feature extraction steps mentioned above.

**Figure 2:**
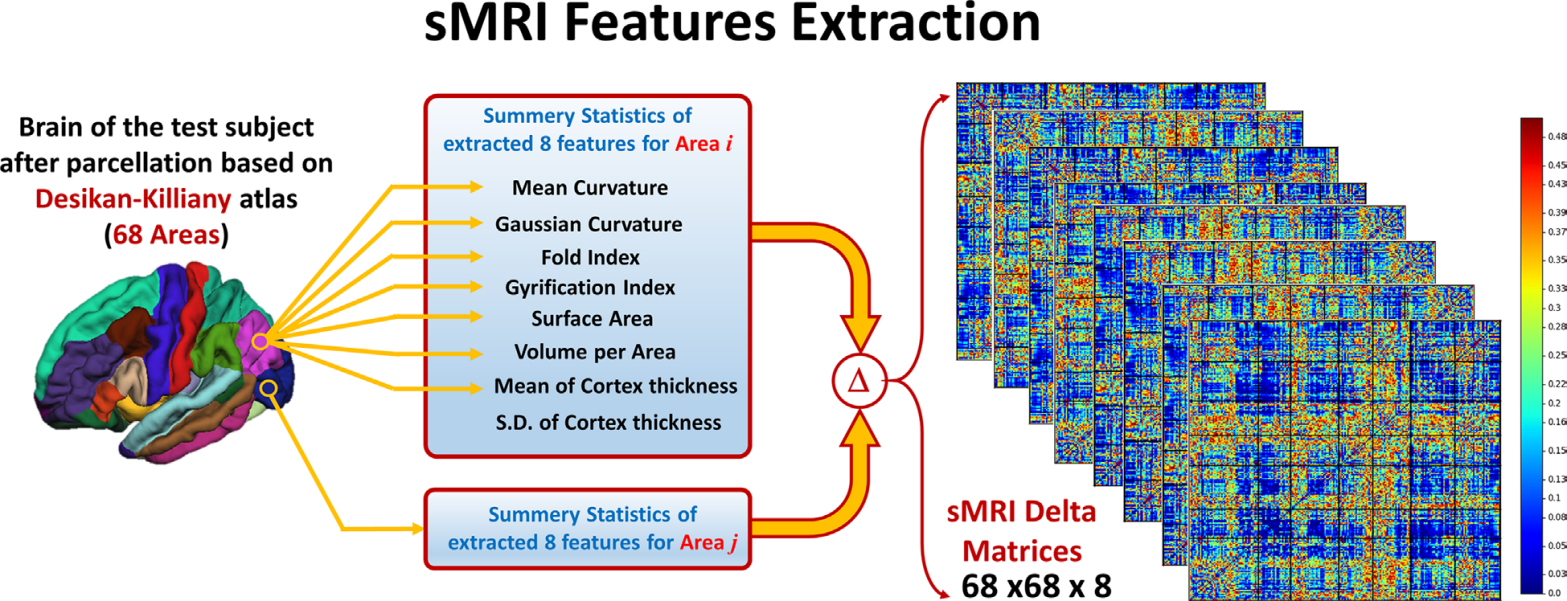
Eight features are extracted from the parcellated volume; summary statistics are calculated for each feature at each DK atlas area. Then, Delta matrix for each subject is calculated by subtracting the feature values between each couple of areas. The output feature matrix is 68 × 68 × 8.

To overcome the problem of variability in the utilized eight features between subjects due to confounding variables like age, IQ, or gender, a normalized form of these features is used. For each subject and for every feature, a 68 areas × 68 areas delta matrix is created, where each element in this matrix is the difference in the feature value between two different areas. In this way, the interaction between the feature values at different areas is studied instead of using the individual feature value per area. Using this technique adds more robustness to the system against variability between subjects due to any confounding variables like age, gender, or IQ. The pipeline of the sMRI experiment is shown in **Figure 2**.

In order to assess how the extracted features are associated with autism severity, a correlation analysis is performed between the difference in feature values among all subjects having ADOS report and the ADOS overall score.

### fMRI Experiment

In the fMRI experiment, the features used are the functional connectivity coefficients between each couple of areas in the DK atlas. The first step in the fMRI analysis is the data preprocessing. The preprocessing in this experiment was applied using the FSL-5.0 nuero-imaging toolbox. The preprocessing steps applied in this study are as follows:

1. Slice timing correction: In an interleaved order, to correct for the effect of acquiring 2D slices at different time shifts.
2. Motion correction (56): To correct for unintended subject motion in the scanner.
3. Normalization to MNI-152 space: The normalization is applied using two-step reregistration. The first step is to register the fMRI subject to its structural image. The second step it to register the sMRI image to the MNI-152 space.
4. Spatial smoothing: A Gaussian filter of full width at half maximum (FWHM) of 6 mm is applied to remove the spatial noise.
5. High-pass filtering: To remove the low-frequency drifts effect.

The main purpose of this experiment is to study the functional connectivity within each subject and how it is capable of diagnosing autism. The functional connectivity was selected to be the used feature as it gives an indication about the coherence of activation between the different brain areas. Hence, it is useful in identifying the brain functional networks and how these networks’ connectivity could be altered between (57) autistic and typically developed subjects. Since we are concerned in this study with DK cortical parcellation, the functional connectivity matrix is constructed between each pair of these atlas areas.

**Figure 3:**
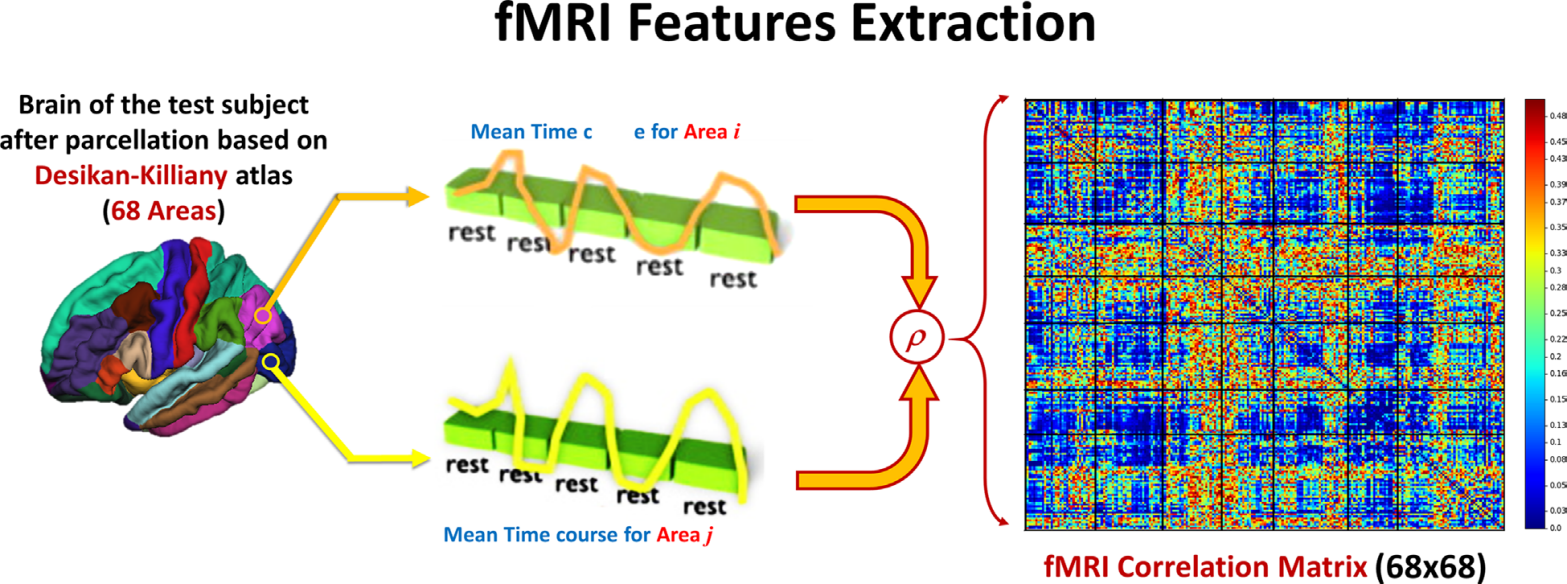
For each couple of areas, the correlation coefficient between the time courses is calculated to form one 68 × 86 feature matrix.

After calculating the preprocessing, the subject 4-D volume is masked with each of the DK atlas areas to calculate the mean time course of this area. The Pearson correlation coefficient (ခρ) is used to calculate the functional connectivity between each pair of areas in the atlas. **Figure 3** shows how feature matrix of the functional connectivity is calculated in this experiment. After calculating the connectivity matrix and assessing how the altered connectivity pattern could reflect autism severity, a regression model is used to fit the functional connectivity coefficients with the overall ADOS severity score in the same way as in the sMRI experiment.

### Local and Global Diagnosis

Having the sMRI and fMRI features ready, the same classification pipeline is applied to both of them. As mentioned above, the fMRI features are a 68 × 68 connectivity matrix, *F* per subject.

(8) $$F=\left[\begin{array}{ccc} \rho_{1,1} & \rho_{1,2} & \cdots \\ \vdots & \ddots & \\ \rho_{n,1} & & \rho_{68,68} \end{array}\right]$$

where ρ*_i,j_* is the Pearson correlation coefficient between time courses in area *i* and area *j* and *n* is the index of areas (*n* = 68). For the sMRI features *S*, they are a 68 × 68 × 8 matrix per subject and each element in the difference in each of the eight features values between each couple of areas.

(9) $$S_{f}=\left[\begin{array}{ccc} \Delta_{1,1} & \Delta_{1,2,f} & \cdots \\ \vdots & \ddots & \\ \Delta_{68,1,f} & & \Delta_{68,68,f} \end{array}\right]$$

where *S* *_f_* is the feature matrix of the feature *f* and *S* *_i,j_* *,* *_f_* is the difference in the values of feature *f* between areas *i* and *j*.

With these feature matrices, one for each modality, a local classifier is applied for each element in each of the two matrices. Both the accuracy and the output probability of each feature to be belonging to the autism class are recorded. The local classifiers used for both sMRI and fMRI are KNN classifiers with number of neighbors = 7. After finishing the local classification phase, the features for each modality are sorted according to the local classification accuracy they achieved in the first step.

KNN is a non-parametric, distance-based classifier. The KNN algorithm assigns a membership score to each new sample based on the number of closest K-neighbors samples from this sample belonging to each of the classes. Based on a majority voting, the sample is assigned to one of the classes (58).

The second step in this diagnosis system is to use the sorted feature vector of both sMRI and fMRI for the per-modality diagnosis. In this step, an incremental approach was used by adding one feature at a time to the used feature vector and recording the cross-validation accuracy until reaching the optimal feature vector length per modality. In this step, a random forest classifier is used. To adjust the hyperparameters of the random forest (number of estimators and maximum depth of the tree), a grid search is used.

Random forest is an ensemble machine learning algorithm combining multiple decision trees using bootstrap aggregating. Each decision tree is fed with a bootstrap of the data with replacement. In order to calculate the feature selection when using random forest, GINI impurity is used (59, 60).

**Figure 4:**
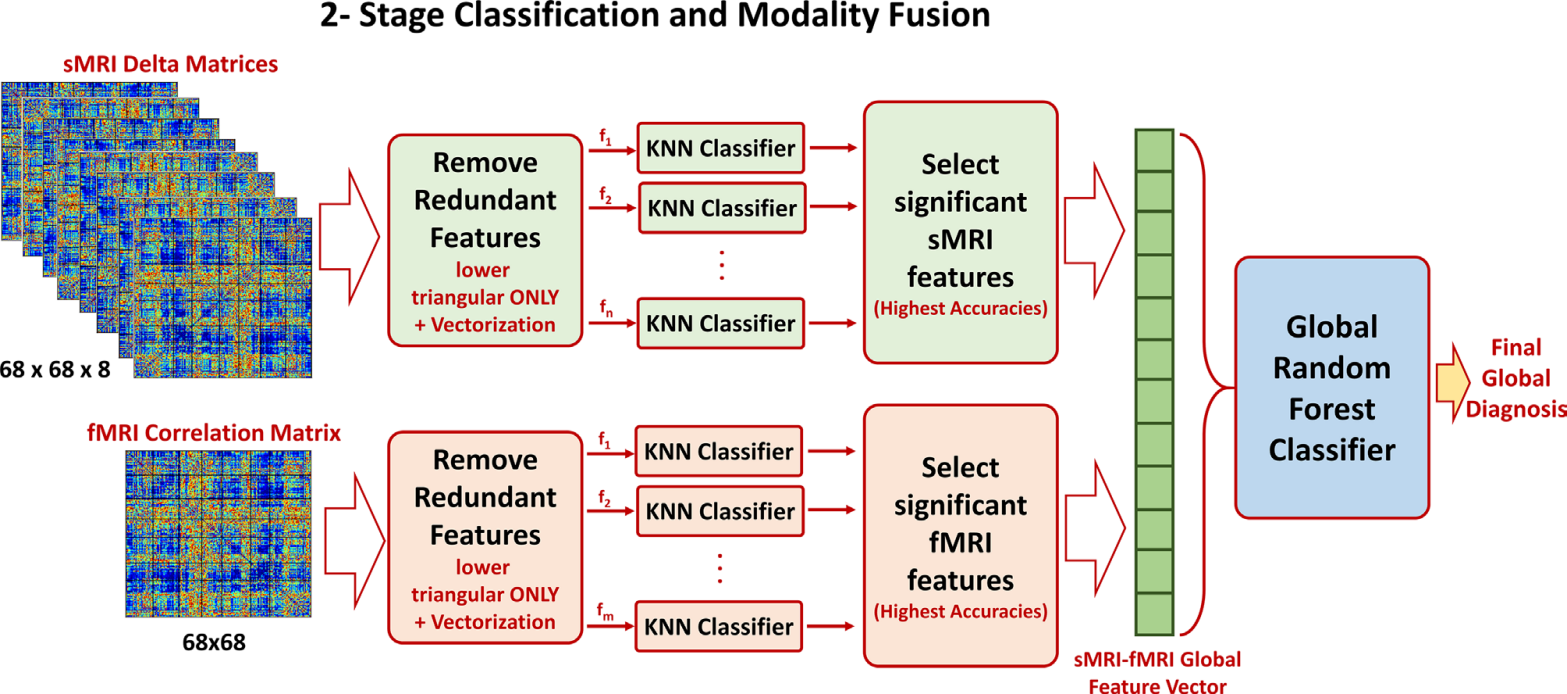
The two-stage classification approach used. In the first stage, a local classification per feature in sMRI and fMRI feature matrices is used. The output accuracies of the first stage are used to create sorted feature vectors. An incremental approach is used to determine the optimal length of the sMRI and fMRI feature vectors. These two vectors are finally concatenated for the global classification.

**Figure 5:**
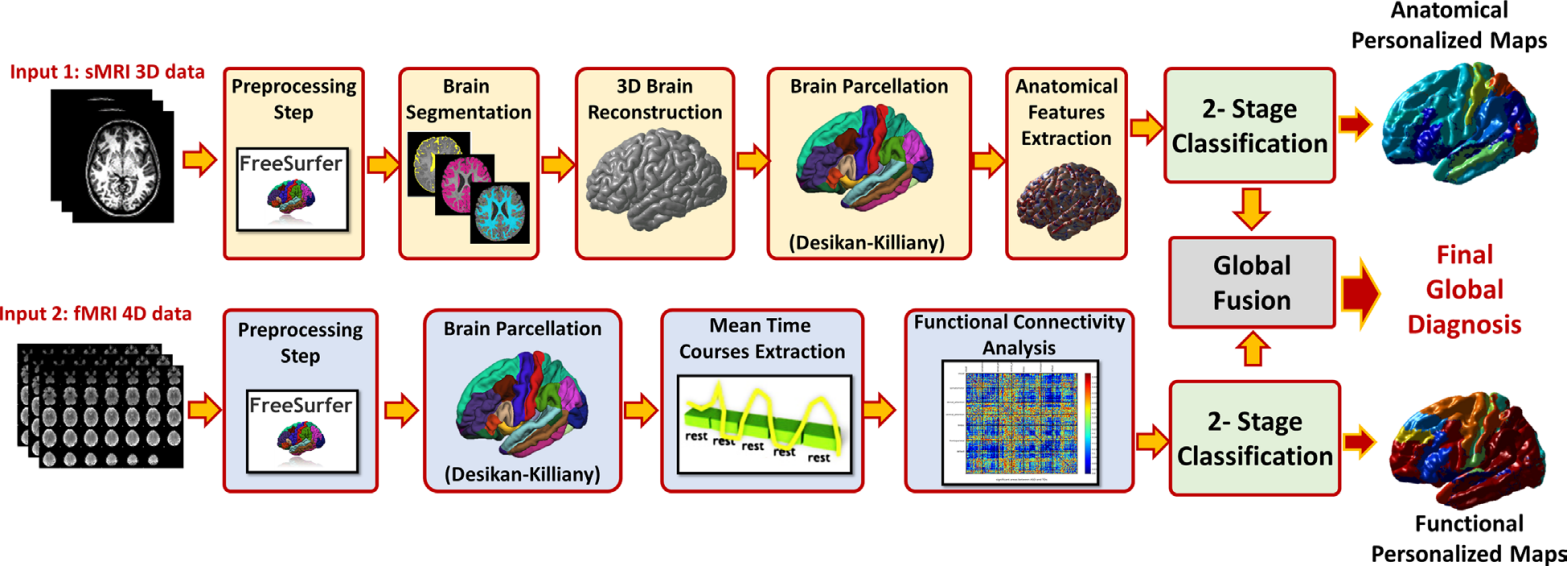
The overall pipeline of the proposed approach, for each modality preprocessing and analysis is applied to calculate the feature matrix. The features of the modality are fed to a local classifier, modality fusion decision is then calculated and finally overall diagnosis decision is reported using both sMRI and fMRI decisions.

Once the optimal cutoff threshold is obtained for each modality and the optimal feature vector is determined for sMRI and fMRI, these two feature vectors are concatenated and fed to another random forest classifier for the global diagnosis decision. The two-step classification approach used is illustrated in **Figure 4**. Also, **Figure 5** illustrates the whole pipeline of the proposed methodology in this study.

## Experimental Results

### Subjects Demographics and Cohort Summary Statistics

Out of the 185 subjects used, 7 subjects were excluded from the fMRI analysis and hence from the sMRI–fMRI fusion because they have corrupted fMRI volumes. The dataset contains 72 autistic subjects (33 males and 39 females) and 113 typically developed subjects (49 males and 64 females). The gender was statistically tested using chi-squared test, and it was found statistically insignificant, χ^2^ = 0.01088, *P* = 0.744. For the ASD group, the males’ mean age is 13.07 years while the females mean age is 13.53. In the TD group, the males’ mean age is 13.04 and the females’ mean age is 12.8125. The age difference between the two groups is also statistically insignificant (*t* = 0.95, *P* = 0.343). The ADOS scores for 61 are available. The social affect (SA) ADOS varied between 0 and 19 with a median of 9, the RRBs varied between 0 and 6 with median of 2.5, while the cumulative ADOS varied between 1 and 24 with a median 11.5. **Table 1** shows the entire summary statistics of the used cohort.

**Table 1:** The used cohort summary statistics.

	ASD		TD		
	Males	Females	Males	Female	
Count	33	39	49	64	*p* = 0.744
Age mean	13.07	13.53	13.04	12.81	*p* = 0.34
Age SD	2.75	2.58	2.68	3.17	
	**Median**	**Range**			
SA	9	0–19			
RRB	2.5	0–6			
Cumulative	11.5	1–24			

### Correlation Analysis With ADOS Total Score

For each of the features used in the two modalities, the correlation between the feature values in the 61 subjects having ADOS overall score and the corresponding ADOS score was studied. The selected correlation thresholded at a correlation of 0.32, which corresponds to a *P* value of 0.01.

In the fMRI experiment, 31 features have correlation coefficients above the significance threshold. In the sMRI experiment, there are 345 features above the selected threshold. The number of features meeting the significance criteria in the sMRI is much higher than that in fMRI as the number of features used in sMRI is eight times the number of features in fMRI.

**Figure 6:**
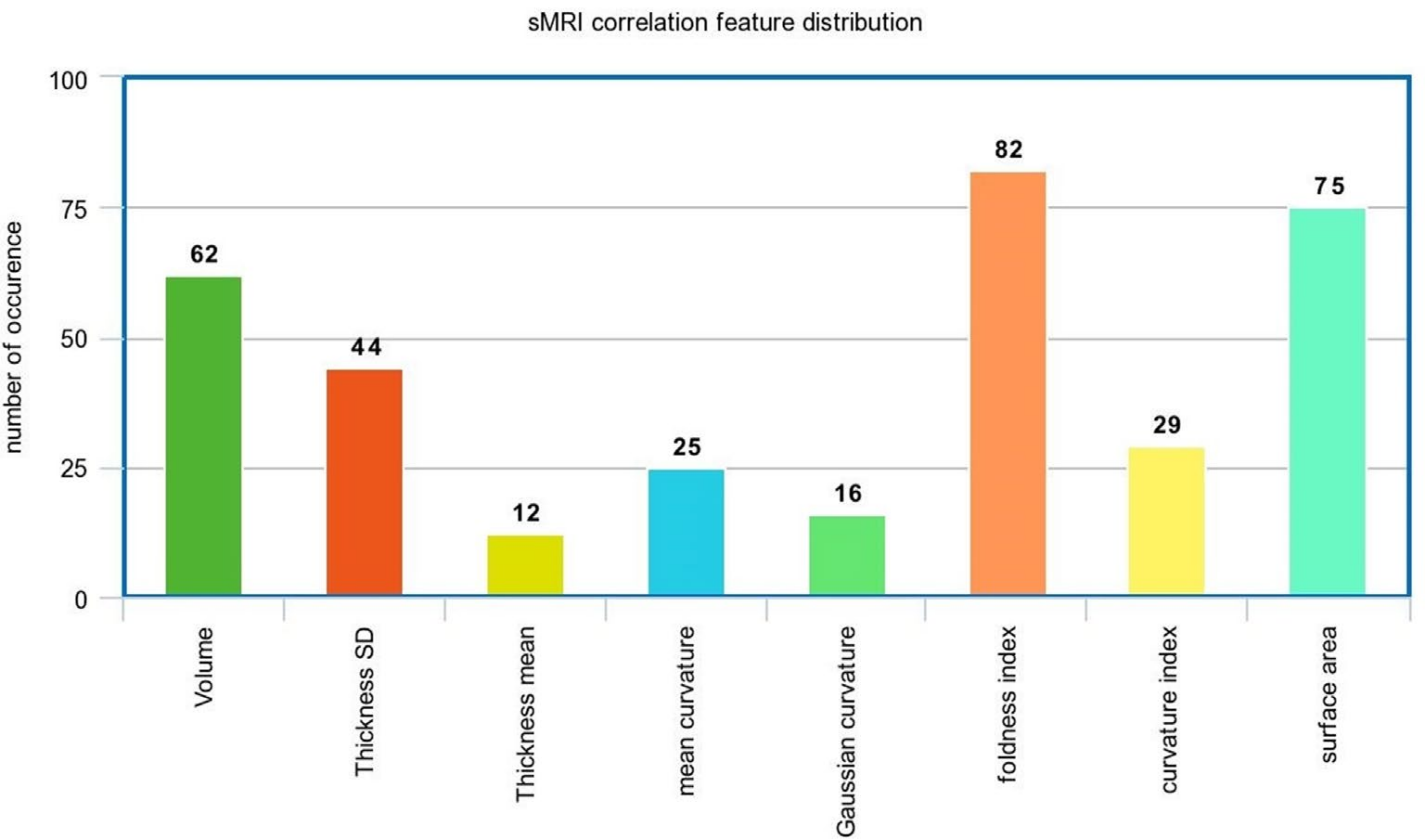
The frequency of occurrence of each of the sMRI features in the significantly correlated features list with ADOS overall score.

**Figure 7:**
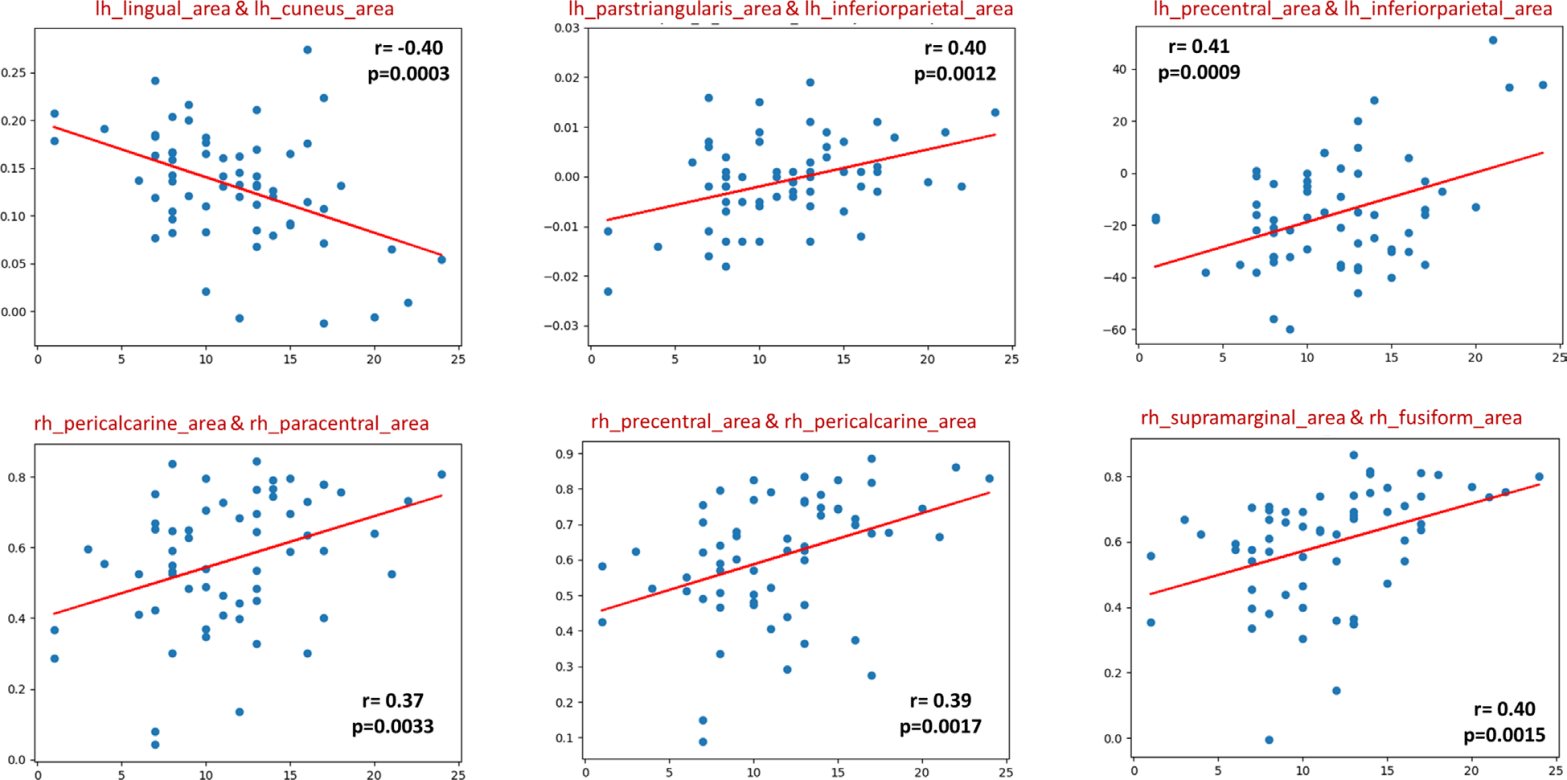
Six selected samples to show the correlation between the feature values in sMRI (upper row), fMRI (lower row), and the ADOS overall score. For each subplot, the figure title shows the couple of area numbers in the DK atlas, the feature name in the case of sMRI, the correlation coefficient, and the *P* value.

**Figure 8:**
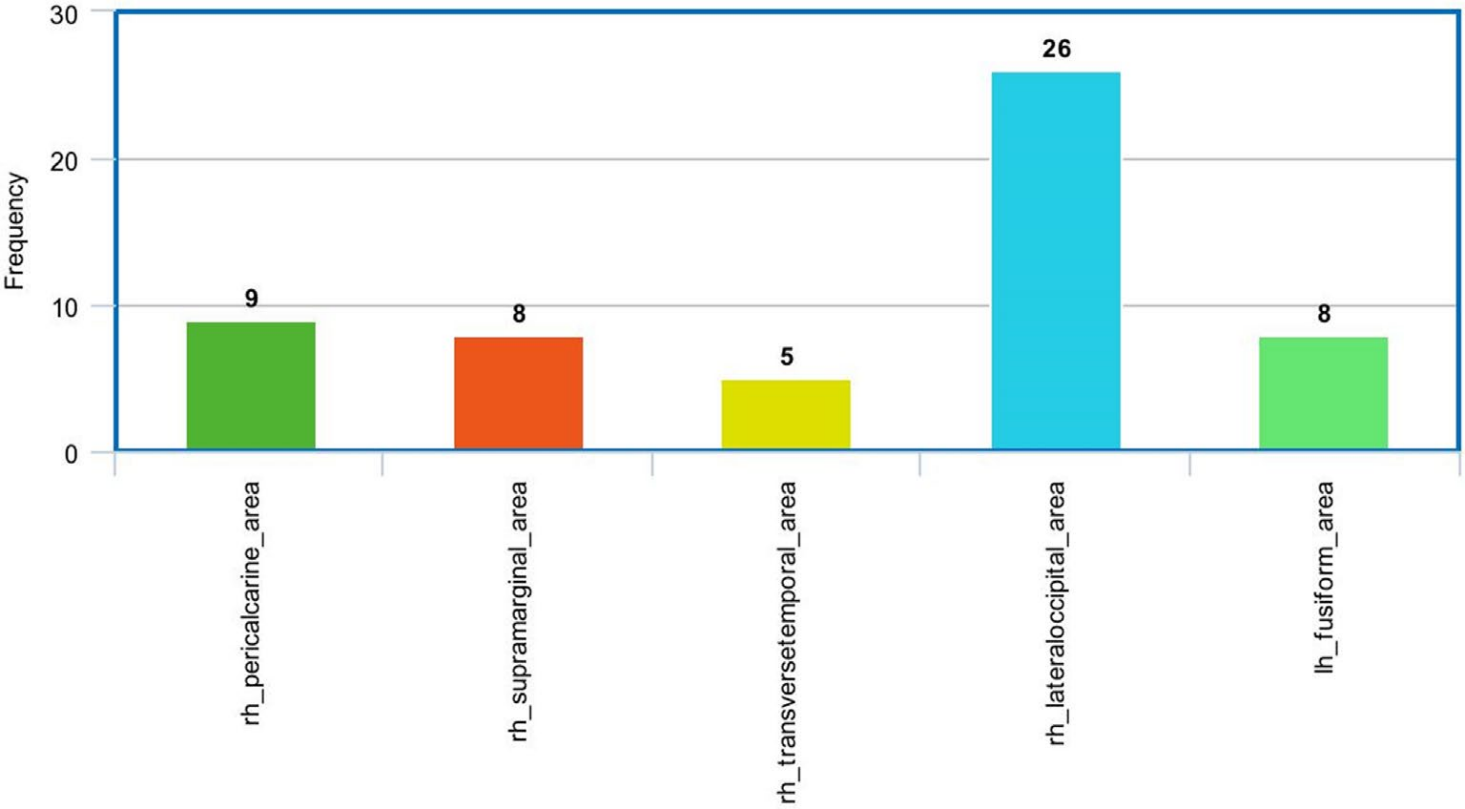
The most frequent areas in the fMRI experiment found to be associated with significantly correlated features with ADOS overall score.

**Figure 9:**
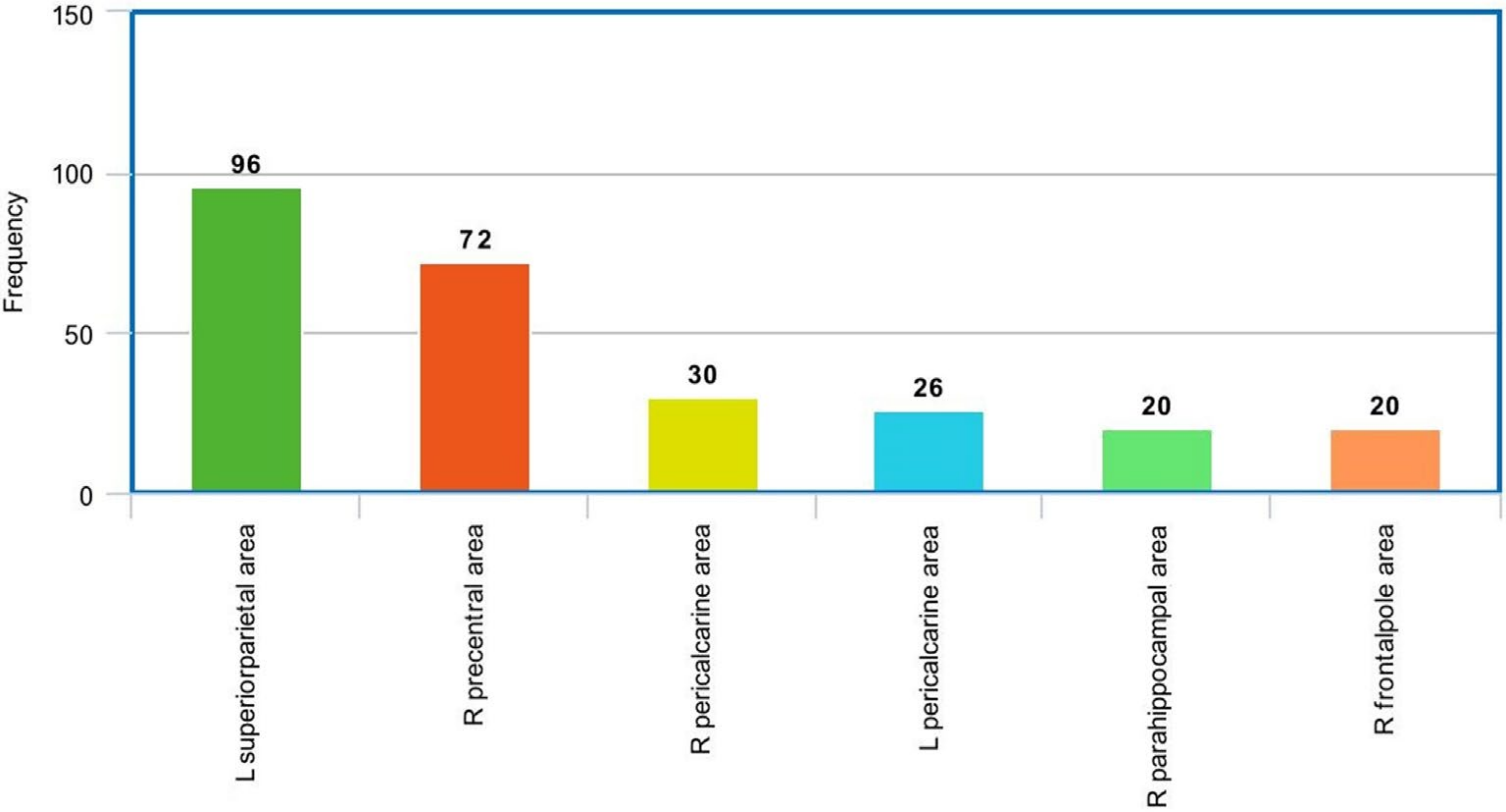
The most frequent areas in the sMRI experiment found to be associated with significantly correlated features with ADOS overall score.

In the sMRI feature matrix, the distribution of the features above the significance level is found to be as follows (**Figure 6**): volume: 62 times, thickness standard deviation: 44 times, thickness: 12 times, mean curvature: 25 times, Gaussian curvature: 16 times, foldness index: 82 times, curvature index: 29 times, and SA: 75 times. **Figure 7** shows sample of correlation of both sMRI and fMRI with ADOS overall score. Also, **Figures 8** and **9** show the most frequent areas associated with features having significantly correlated features with overall ADOS score.

### Local and Global Diagnosis

For each subject, the local probabilities for both sMRI and fMRI feature matrices are calculated. These probabilities are used to generate the personalized brain maps. The output of the local classification is two matrices *P* *_S_* and *P* *_F_* with the same size as the feature matrices *F* and *S*.

(10) $$P_{F}=\left[\begin{array}{ccc} pf_{1,1} & pf1,2 & \cdots \\ \vdots & \ddots & \\ pf & & pf68,68 \end{array}\right]$$

(11) $$P_{S}f=\left[\begin{array}{ccc} Ps_{1,1,f} & Ps_{1,2,f} & \cdots \\ \vdots & \ddots & \\ Ps_{68,1,f} & & Ps_{68,68,f} \end{array}\right]$$

where *pf* *_ij_* if the probability of the functional connectivity between areas *i* and *j* to belong to the autism class and *Ps* *_i,j,f_* is the probability that the difference in the structural feature *f* between areas *i* and *j* belong to the autism class.

To obtain the personalized maps, two vectors, *V* *_f_* and *V* *_s_*, are calculated for fMRI and sMRI, respectively.

(12) $$V_{f}(i)=\underset{0\leq j\leq 68}{\max}P_{F}(i,j)$$

(13) $$V_{s}(i)=\underset{i\leq j\leq 68,1\leq f\leq 8}{\max}P_{S}(i,j,f)$$

**Figure 10:**
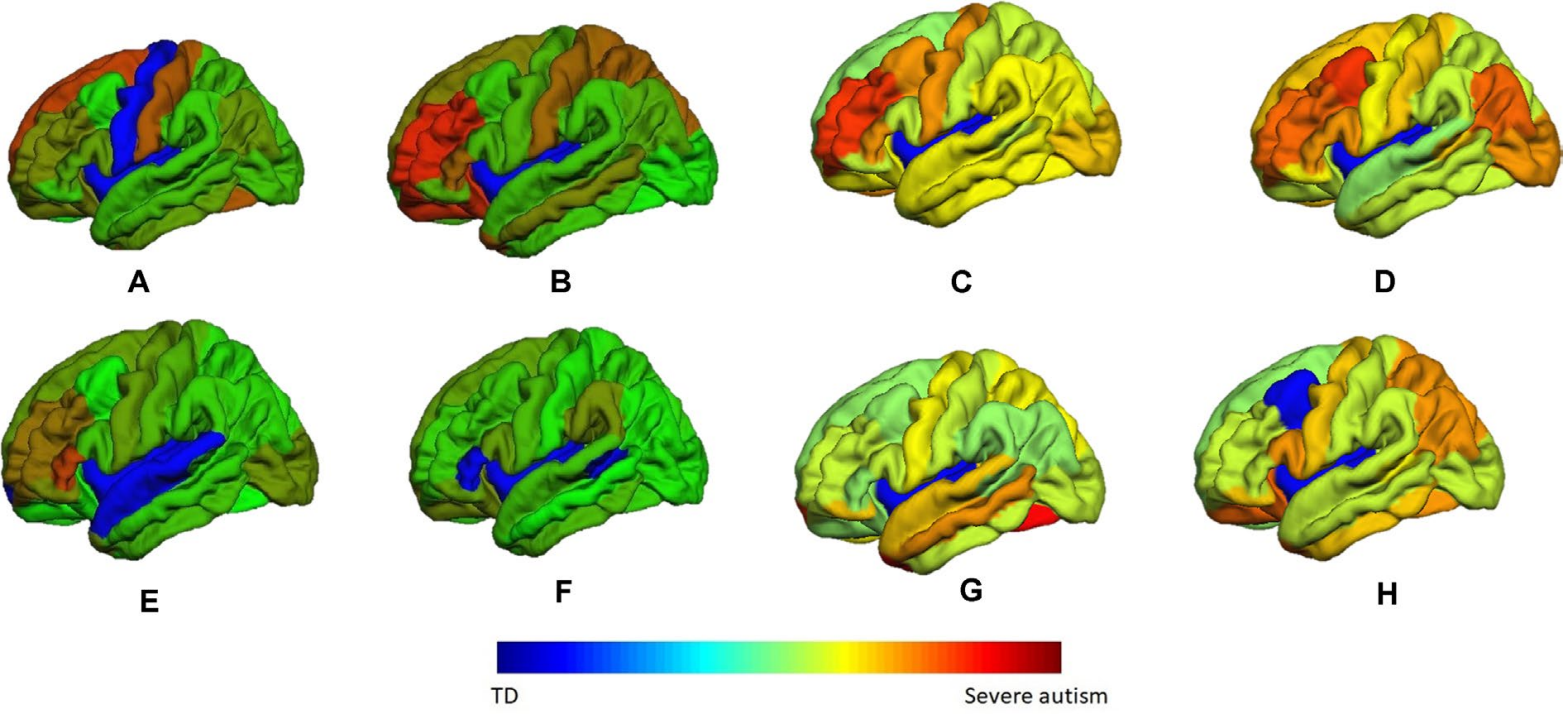
A sample of the generated personalized maps for eight subjects: **(A, B)** are the personalized maps of two ASD subjects obtained from sMRI local classification, **(C, D)** are the personalized maps of two ASD subjects obtained from fMRI local classification, **(E, F)** are the personalized maps of two TD subjects obtained from sMRI local classification, and **(G, H)** are the personalized maps of two TD subjects obtained from fMRI local classification.

These two vectors indicate the highest probability of an area to be belonging to the autism class. A sample of these color coded maps is shown in **Figure 10**.

### Per-Modality Diagnosis Results

**Figure 11:**
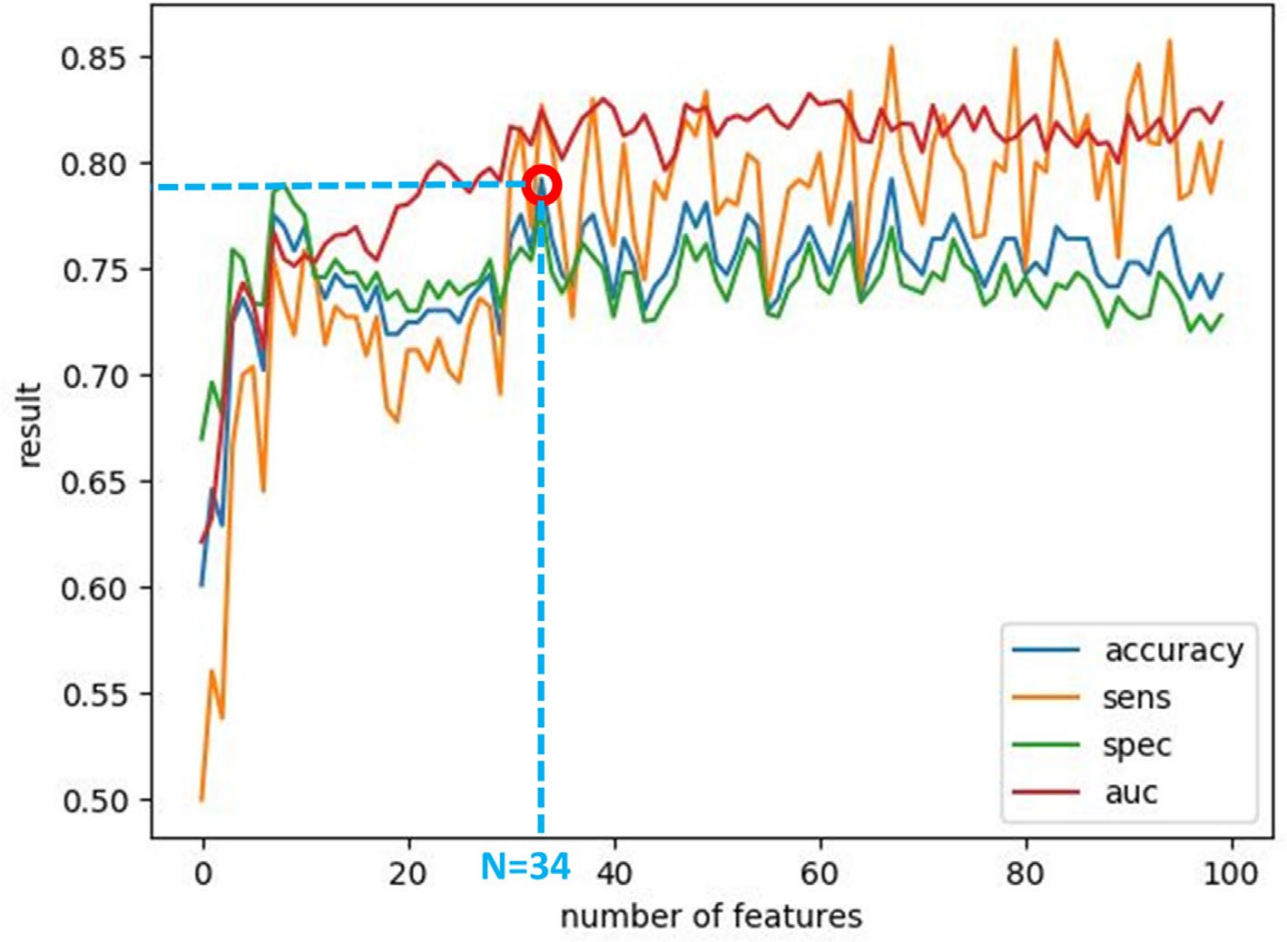
The accuracy, sensitivity, specificity, and AUC obtained by changing the number of selected features for sMRI between 1 and 100 features. The maximum accuracy achieved is obtained when using 34 features.

**Figure 12:**
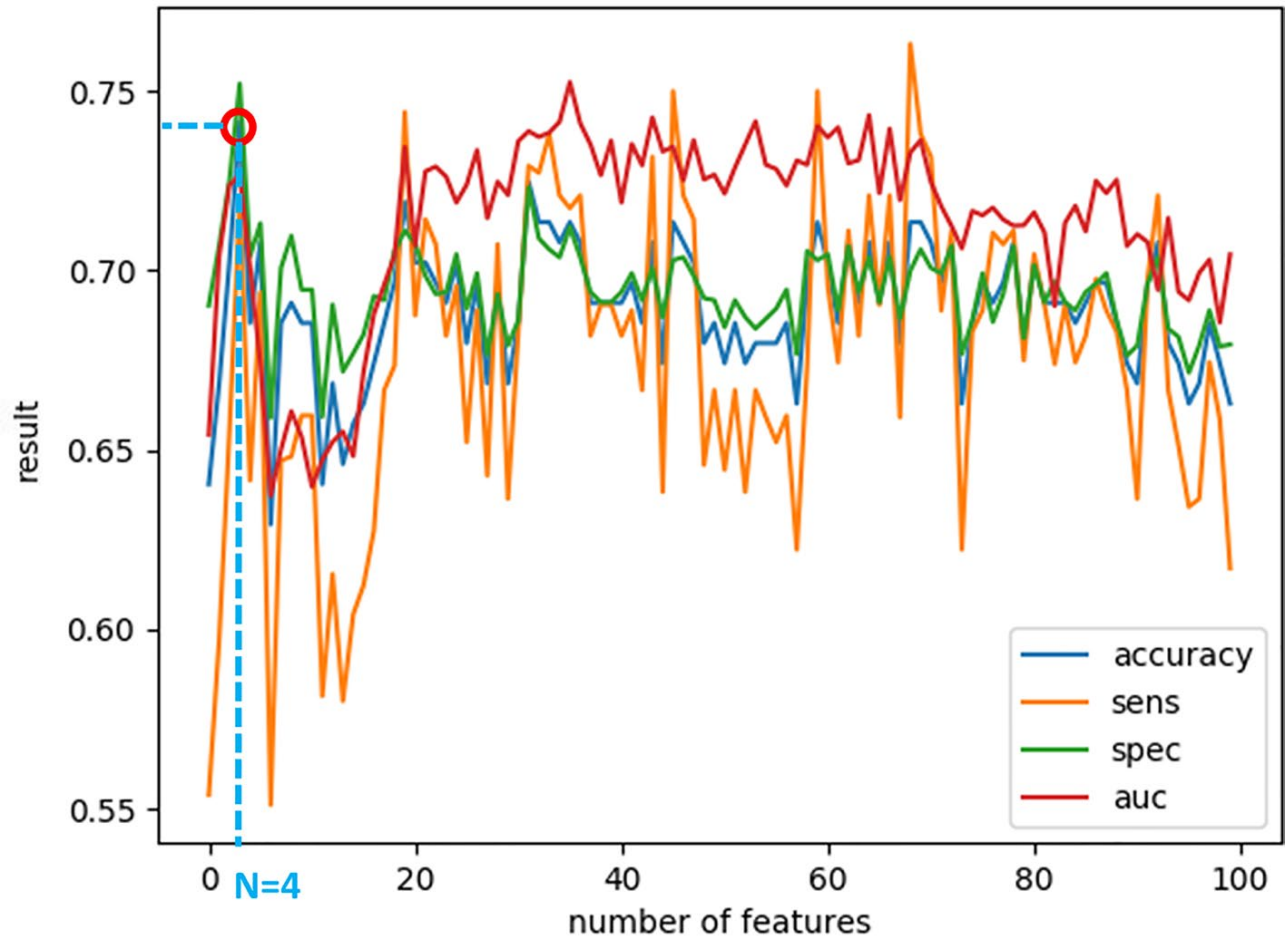
The accuracy, sensitivity, specificity, and AUC obtained by changing the number of selected features for fMRI between 1 and 100 features. The maximum accuracy achieved is obtained when using four features.

After calculating the local probabilities, *P* *_S_* and *P* *_F_*, they were sorted according to the obtained accuracy. A linear scan is done to find the optimal number of features to concatenate for sMRI and fMRI. From this scan, it is found that by fusing the first 34 sorted features in sMRI and the first 4 features, the highest sMRI and fMRI accuracies are achieved. To show the effect of changing the number of selected features, **Figures 11** and **12** show the accuracy, sensitivity, specificity, and area under the curve (AUC) when using different numbers of features (from 1 to 100 areas). **Table 2** shows the accuracy, sensitivity, specificity, and AUC obtained for sMRI and fMRI when selecting the first 34 and 4 features, respectively. The reported results used random forest classifier with fourfold cross validation.

**Table 2:** The comparison between random forest, SVM, naive Bayes, and neural network results for the global fusion.

	sMRI	fMRI
**Accuracy**	0.79	0.74
**Sensitivity**	0.82	0.72
**Specificity**	0.77	0.75
**AUC**	0.824	0.72

### Global Diagnosis Results

After knowing the optimal number of features to be used from both sMRI and fMRI, these features are concatenated together to form a global feature vector. The output global feature vector contains 38 features. These 38 features are then fed to a random forest classifier to obtain global accuracy, sensitivity, specificity, and AUC. These results are 80.8%, 84.9%, 79.2%, and 81.92% for the accuracy, sensitivity, specificity, and AUC, respectively. In addition, a comparison between different classifiers in the global diagnosis is reported in **Table 3**.

**Table 3:** The accuracy, sensitivity, specificity, and AUC obtained for sMRI and fMRI when selecting the first 34 and 4 features, respectively.

	Random Forest	SVM	Naïve Bayes	Neural network
**Accuracy**	0.808	0.71	0.75	0.68
**Sensitivity**	0.849	0.72	0.77	0.7
**Specificity**	0.792	0.67	0.71	0.64
**AUC**	0.819	0.73	0.73	0.67

## Discussion

The challenge of understanding the child’s individual neural circuitry is daunting. Multiple reports in general suggest hypoconnectivity (35) in most studies. However, neurophysiological and MRI evidence does suggest local hyperconnectivity in some brain regions (28). This report extends our previous fMRI findings (61–63) and suggests that particular MRI parameters related to the expanded neuropil in mini-columns including foldness index, SA, and volume are more relevant to defining ASD-related neural circuits (64). These parameters make it possible to link between two adjacent Brodmann areas (BAs) or brain regions, which directly increase the correlation to behavior. Again, the local diagnosis of our algorithm identified ASD-related brain regions that fit into RDoC neural circuits and are similar circuits found to be predictive of ASD diagnosis at 24 months.

### Computer-Aided Diagnostic System for ASD

The current dataset suggests that it is possible to define a localized diagnosis, which is the key to defining each relevant ASD neurocircuit within an individual. The algorithm provides high accuracy, sensitivity, and specificity when sMRI or fMRI are analyzed separately. Our current algorithm also fuses the sMRI and fMRI datasets, which provides a greater estimate of 80% accuracy, 85% sensitivity, and 79% specificity. The ability of the algorithm to estimate a whole-brain diagnosis was validated by a cross-validation technique using fourfolds. The principle of dataset fusion is to handle the individual variability of brain structure and function that is impacted by various genetic and environmental factors.

Further, the principle of data fusion by Big Data techniques will delineate key circuits that correlate with behavioral output of those circuits. Our careful approach has studied the different MRI parameters that are inputs into the machine learning algorithm. While it may be possible that, as a whole, global diagnosis may be better *via* fMRI vs. sMRI, our study suggests that the addition of fMRI or sMRI parameters in regions specific for the ASD diagnosis/classification of regions linked in neural circuits gives an even higher significant Pearson correlation (0.37–0.45) at *P* = 0.001 than previous Rs-MRI (0.25) (62) data with ADOS total score. Similarly to what was found to be predictive in high-risk infants who then developed ASD at 24 months, we found that MRI parameters related to the neuropil expansion (volume, SA, and folding index) and fMRI parameters (functional connectivity measure) were features that mediated the significant Pearson correlation between any two brain regions and ADOS total scores. These significant correlations between brain regions were most significantly frequent in the posterior brain regions. Such observations would be consistent with the overall increased functional connectivity observed in the posterior cortex (28). Whether the predominance of right hemispheric correlation over left hemispheric correlation is significant will await the input of further data. Thus, the current data suggest that the approach of a localized diagnosis with fusion of multi-model datasets will greatly improve accuracy, sensitivity, and specificity while linking two or more adjacent BAs or brain regions to directly increase the correlation to behavior.

## Limitations of the Approach

While neuroimaging is an attractive and easily obtainable piece of clinical data, the experiments here are limited by different sources of data including harmonization of scans for head motion, different MRI scanners and sequences, plus fMRI data obtained under different conditions. Such variables could limit the utility of our data in building a personalized medicine model. Further the drawback of the current data and MRI methods includes defining the developmental trajectory, impact of age/gender, development of clinically applicable techniques for scanning across ages, and the unknown nature of the relationship between modern psychology diagnostics/behavioral testing and MRI/genetic data. The current findings may be only applicable to older ages (8–18 years old) and higher-functioning ASD subjects. However, the current data link multiple BA regions in RDoC neurocircuits implicated in ASD, thereby suggesting the scalability of this approach to larger, more heterogeneous ASD populations.

The lack of longitudinal fMRI data in the under 8-year population of typically developing children may limit the approach (65). The number, diversity of subject pool (age/gender), design of MRI protocols, and preprocessing/methods of analyses are still variables under study. Additionally, the methods for analyses and selections of datasets for our machine learning algorithms are still not standardized and must yield biologically relevant information. The generalization and feasibility of a system will be improved by increasing the number of subjects and the intra-variability between subjects, including age/sex, multiple scanner data, and other factors.

## Future Directions: Multi-Model Fusion Links to RDOC Neurocircuits

Our previous data using fMRI alone from the NDAR dataset identified frontopolar and temporal parietal junction functional networks as key regions correlated with ADOS scores and mapped to RDoC neurocircuits (62). The new extended list of ADOS-associated brain regions (pericalcarine cortex, lateral occipital cortex, supramarginal gyrus, transverse temporal, fusiform gyrus, superior parietal, precentral gyrus, parahippocampal gyrus, and frontal polar regions) includes the previous regions but greatly expands the RDoC neurocircuits identified through as localized diagnosis in ASD brains (65).

The greater mapping of behaviors over an anatomical and functionally linked circuit is more likely to map a cluster of ASD individuals whose behaviors and characteristics are more similar than different. Further, the division of behavioral clusters across specific neurocircuits may identify not only traditional autism susceptibility genes like those in the SFARI database but also ASD modifier genes that subtly impact the structure and function of circuits, thereby influencing behavioral output of the circuit. Such ASD modifier genes (in the genetic background of an individual) may mediate the gene–environment interactions responsible for 50% of ASD etiology. This group of genes may be quite large but have low effect size and therefore would not be picked up in traditional autism genetic studies (66). This group of genes may be represented among the transcriptome studies reported in ASD where altered transcripts may not necessarily correspond to a specific autism susceptibility gene but may define specific developmental trajectories (42). Such genes or traditional ASD susceptibility genes may also define specific MRI clusters. In a recent report (67), three groups of MRI phenotypic clusters were defined during an fMRI survey of mouse models of autism. Group 1 (SGSH, TREM1, FMR1, and CNT2) had hypoconnectivity involving the PFC, BG, retrosplenium of CC, and thalamus plus hyperconnectivity of the ventral striatum/nucleus accumbens. Group 2 (CDKL5, EN2, MECP2, and CHD8) had whole cortex/BG hypoconnectivity plus increased functional connectivity of the lateral septum. Group 3 (Syn2, BTBR, and 16p11 deletion) had increased functional connectivity in PFC, insula, parietal cortex, amygdala, midbrain and hypoconnectivity in sensory cortex, ventral striatum, and thalamus. Further, some mutations (EN2, FMR1, MECP2, and SGSH) had highly correlated changes between hemispheres while other mutations had a low correlation between hemispheres (16p11 deletion and Syn2). The MRI evidence supports the hypothesis that multiple behavioral clusters may map onto distinct MRI phenotypes involving the frontal, temporal, and parietal cortices identified here as well as important subcortical structures (nucleus accumbens, striatum, and thalamus). While distinct ASD susceptibility genes may define global MRI phenotypes, our careful comparisons of fused datasets with genomics are likely to identify more subtle modifier ASD genes that finally influence the local sculpting and function of neural circuits during specific developmental periods, thereby producing more distinct behavioral clusters. In summary, the advancement of new technologies in psychology, radiology, and genetics has allowed never before interrogation of datasets that further delineate human biology. The use of Big Data technology is the only current realistic experimental methodology that could define the variability of neural circuits and linking genetics with behavior in such a polygenic disease such as ASD. This study demonstrates that fusion of MRI data and machine learning could refine diagnostic accuracy, especially at the local neurocircuit level. Such data could define clinically distinct endophenotypes from particular affected neural networks and therefore amenable to targeted pharmacological and/or behavioral interventions. The goal of this project is to ultimately develop personalized treatments for ASD. The next phase of this study will focus on the full integration of genomic, behavioral, and MRI datasets to further define the feasibility, robustness, and generalizability of our systems. In addition, more data will be included for subjects at younger ages and infants; also, some other phenotypes, like ADHD for example, will be included. In this way, the system will be more comprehensive with higher diagnosis ability for ASD.

## Author Contributions

OD has the primary responsibility for the conduct of the research and wrote the bulk of the manuscript. MA, YE-N, and AM contributed to the guidance in data collection and preparation and manuscript writing. ASh contributed to the guidance in data preparation, manuscript writing, and figure preparation. ASo contributed to the guidance in sMRI experiment, manuscript writing, and figure preparation. ASw performed statistical analyses and reporting, and contributed to the guidance in algorithm validation and results interpretation. MG and HH contributed to the guidance in fMRI experiment and guidance in literature review and manuscript writing. MC contributed to the guidance in medical-related points and was responsible for results interpretation and validation. AE and RK contributed to the supervision, manuscript revision and editing, and results interpretation and verification. AE-B contributed to the project initiation and idea preparation, supervision, manuscript revision, and editing and results interpretation and verification. GB, the medical collaborator, guided in all medical-related points and also was responsible for results interpretation and validation. He was also responsible for the writing, editing, and revision of the manuscript.

## Funding

This work has been supported by the University of Louisville 21st Century University Initiative on Big Data in Medicine.

## Conflict of Interest Statement

The authors declare that the research was conducted in the absence of any commercial or financial relationships that could be construed as a potential conflict of interest.
